# Thermal Mechanisms Preventing or Favoring Multiple Ovulations in Dairy Cattle

**DOI:** 10.3390/ani11020435

**Published:** 2021-02-08

**Authors:** Fabio De Rensis, Giorgio Morini, Irina Garcia-Ispierto, Fernando López-Gatius

**Affiliations:** 1 Department of Veterinary-Medical Science, University of Parma, Str. dell’Università, 12, 43121 Parma, Italy; fabio.derensis@unipr.it (F.D.R.); giorgio.morini@gmail.com (G.M.); 2 Department of Animal Science, University of Lleida, 25198 Lleida, Spain; irina.garcia@udl.cat; 3 Agrotecnio Centre, 25198 Lleida, Spain; 4 Transfer in Bovine Reproduction SLu, 22300 Barbastro, Spain

**Keywords:** graafian follicle, ovarian physiology, ovulation failure, unilateral ovulations

## Abstract

**Simple Summary:**

While cows are usually monovular, the incidence of dizygous twin births has recently increased considerably alongside increasing milk production. Genetic progress and improvements in nutrition and management practices have led to a continuous increase in milk yield and thus also to multiple ovulations and twin pregnancies. Twin pregnancies are undesirable as they seriously compromise the welfare of the cow and herd economy. A better understanding of the processes associated with multiple ovulations should help to reduce rates of twinning. During the stages of the sexual cycle, temperature gradients are established within the ovary and throughout the genital tract. Pre-ovulatory local cooling of the reproductive system favors male and female gamete maturation and subsequent fertilization. In fact, thermal mechanisms may prevent or favor multiple ovulations and thus twinning. The purpose of this review was to update this topic.

**Abstract:**

While cows are predominantly monovular, over the past 30 years the incidence of multiple ovulations and thus twinning has increased considerably alongside milk production. Multiple pregnancies are not desirable as they negatively affect the health of cows and the herd economy. Although causal mechanisms associated with multiple ovulations have been extensively revised, the process of multiple ovulations is not well understood. Recent studies on the thermal biology of the reproductive system have shown how thermal mechanisms may prevent or favor multiple ovulations. This review focuses on this relationship between thermal dynamics and multiple pregnancies. Cooling of the pre-ovulatory follicle is able to regulate ovulation. In effect, pre-ovulatory local cooling of the female reproductive system favors male and female gamete maturation and promotes fertilization. Thermal stress is proposed here as a model of stress. Periods of high ambient temperature affect the processes of pre-ovulatory follicular cooling and multiple ovulations. While the ratio between unilateral and bilateral multiple pregnancies is normally close to one, under heat stress conditions, this ratio may be 1.4 favoring unilateral multiple pregnancies. A ratio approaching unity is here proposed as an indicator of cow wellbeing.

## 1. Introduction

Cows are predominantly a monovular species. However, over the last 30 years, the incidence of multiple ovulations and thus twinning in dairy cows has increased considerably alongside milk production [1,2,3,4]. Multiple ovulations are likely partly a consequence of gene selection for milk yield [5]. However, aside from genetic progress, improvements in nutrition and management practices have also led to a continuous increase in milk yield and so to multiple ovulations [6,7,8]. In addition, breeding synchronization protocols for fixed-time artificial insemination (FTAI) have become routine components of the reproductive management of dairy herds. While some short protocols seem to reduce the double ovulation rate compared to spontaneous estrus [9] or to longer protocols [10], the hormone combinations used for FTAI can increase the risk of double ovulation [11,12]. Hence, over the years to come, the multiple ovulation rate will probably continue rising along with milk production. Multiple ovulations and subsequent multiple pregnancies are not desirable as they negatively affect the health of cows and the herd economy [1,2,3,4]. A better understanding of the processes associated with multiple ovulations may help reduce twinning rates. Factors associated with multiple ovulations have been extensively reviewed [4,11,13,14]. However, the causal mechanisms of multiple ovulations are not well understood [14]. Some thermal mechanisms that may prevent or favor multiple ovulations have been recently identified. The purpose of this paper was to update the literature data available on this topic.

## 2. Temperature Gradients in Mammalian Female Reproductive Organs

Pre-ovulatory local cooling of the female reproductive system promotes male and female gamete maturation and plays a critical role in the events of fertilization [15,16,17,18,19]. The temperature within the genital tract increases progressively from the vagina to the utero-tubal junction [20]. Thus, sperm pass from the warmer uterus through the utero-tubal junction into the cooler isthmus [15,16,19], or caudal portion of the oviduct, where their motility is reduced thereby serving as a functional sperm reservoir [21,22,23]. Close to the time of ovulation, spermatozoa are released from the caudal isthmus to the warmer site of fertilization [24]. Thus, temperature gradients promote sperm transport within the mammalian female genital tract [15,16,19]. In fact, temperature differences within the tubular genital organs may well remain as a vestige of terrestrialization, reflecting the transition from external (aquatic) fertilization to the more efficient internal fertilization [25]. The oviducts and uterus provide different aqueous luminal microenvironments for gamete transport [26].

In ovarian tissues, temperature within the follicular antrum during the pre-ovulatory period is able to regulate ovulation [27]. Pre-ovulatory follicles may be over 1 °C cooler than neighboring tissues and both compartments are cooler than rectal temperatures in sheep [28], rabbits [29], women [30], pigs [31,32], and cows [33,34,35,36]. In lactating dairy cows, it has been recently shown that most follicles of pre-ovulatory size that were cooler than deep rectal temperature ovulated, whereas follicles not showing such a temperature differential did not [34,35,36]. In agreement with earlier results in humans [30], the extent of follicular cooling in cows could be positively correlated with the occurrence of pregnancy [36]. In essence, the ovaries may require a lower temperature than neighboring organs to maintain their functions [37], and mature Graafian follicles are always cooler than stroma [15].

Counter-current exchange systems of heat between veins and arteries can set up such temperature gradients. Local transfer of heat has been detected between many organs and is well-established in the reproductive tissues of female mammals [38,39,40]. Local counter-current transfer can be considered a regulation mechanism of organ function that enables thermal and endocrine interplay between ovary, oviduct, and uterus [25].

## 3. Single versus Double Ovulation

The effects of heat stress on the reproductive success of dairy cows have been extensively described [41,42,43]. As local cooling of the reproductive system is the result of counter-current transfer of heat, high ambient temperatures added to the metabolic stress of milk production can compromise ovulation [44]. We thus propose thermal stress as a model for any type of stress affecting pre-ovulatory follicular cooling and multiple ovulations. In effect, global warming is likely already having a negative impact on reproductive functions in mammals [45].

**Figure 1:**
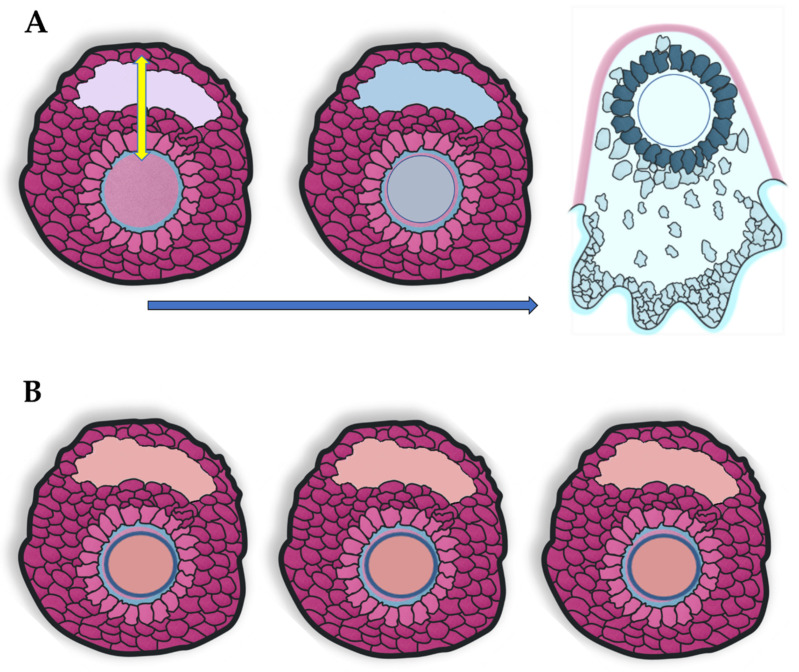
Sequence diagram showing: (**A**) events from a pre-ovulatory follicle undergoing cooling to the process of ovulation, or (**B**) a follicle of pre-ovulatory size not experiencing cooling which fails to ovulate, for example under thermal stress conditions. Color change from light red (pre-ovulatory follicle before cooling) to blue (preovulatory follicle going through cooling) indicates a decrease in temperature in the ovulating follicle. The double arrow indicates the crosstalk between oocyte and somatic follicular cells [25]. Drawing by López-Gatius. The color artwork is courtesy of Cris Segú Mora.

Ovarian follicles of pre-ovulatory size are particularly sensitive to thermal stress. When cows in estrus were exposed over a brief period (<45 min) to high environmental temperatures, they failed to ovulate [34]. The temperature of the non-ovulating follicles was equal to or higher than that of corresponding deep rectal temperatures, whereas follicles of similar size that did sustain cooling ovulated (Figure 1) [34,35,36]. This explains why, in a previous study on cows showing spontaneous estrus, the incidence of ovulation failure and double ovulation were significantly higher and significantly lower, respectively, during the warm than during the cool period [8]. Based on odds ratios, reaching estrus during the warm period increased the likelihood of ovulation failure by a factor of 3.9, whereas it decreased the likelihood of double ovulation by a factor of 0.86 [8]. It is clear that cows not under heat stress during the cool period had a larger number of mature follicles responsive to the process of cooling.

The incidence of multiple ovulations in high producers may be over 20% [6,7,8] or even 30% when following some FTAI protocols [46,47]. However, the multiple ovulation rate is not a true reflection of a higher rate of follicles of pre-ovulatory size (co-dominant follicles) at estrus. Obviously, the presence of two or more follicles is the basis of multiple ovulations. In a recent study on 622 lactating dairy cows, at the time of FTAI, 306 (49.2%) had a single follicle, 198 (31.8%) had two bilateral follicles (one follicle per ovary), and 118 (19%) had two unilateral follicles (same ovary) [48]. Although heat stress at AI (maximum temperature-humidity index > 72) had not effects on follicular dynamics, cows with two unilateral follicles showed a higher double ovulation rate (48.6%) than cows with two bilateral follicles (34.8%) [48]. Inter- and intra-ovarian gradients in temperature and cooling in the same ovary made the ovulation of both unilateral rather than of bi-lateral co-dominant follicles more likely. In the same study, all cows with three or more follicles (*n* = 13) were withdrawn from the experiment [48]. This meant that about half of the cows had two follicles or more at AI, and the subsequent percentage of double ovulations for all cows was 18.2%. This incidence of double ovulation is within the ranges of 12–30% provided by the different studies following FTAI [6,8,46,47,48,49,50], or 15–23% for cows following spontaneous estrus [7,8,51,52].

**Figure 2:**
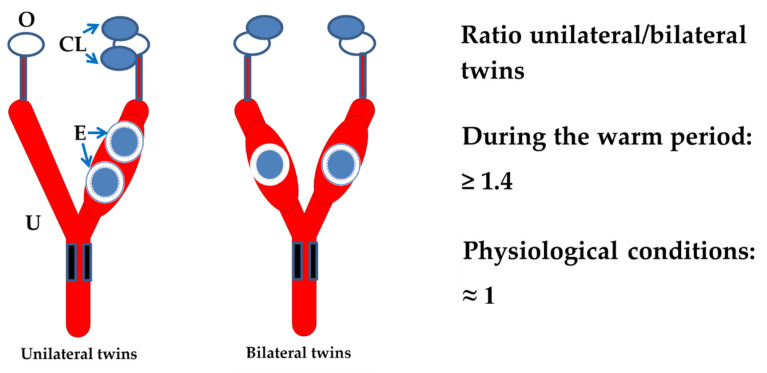
Diagram showing the ratio unilateral/bilateral multiple pregnancies during warm and cool periods [53]. Data from the cool period were considered equivalent to those of normal physiological conditions such that a ratio close to unity could be an indicator of cow wellbeing. As the incidence of triplets and quadruplets was very low, only twins are represented in the figure with their corresponding corpora lutea. O: ovary; CL: corpora lutea; E: embryos; U: uterus.

Data derived from a more recent study reinforce these findings [53]. Incidence patterns of multiple pregnancies (*n* = 1130) were examined in cows becoming pregnant following their third parturition or more and in their partners with one single embryo (*n* = 3160). The cows did not receive hormone treatments before pregnancy. The percentage of unilateral multiple pregnancies (all embryos in the same uterine horn) was significantly higher than that of bilateral pregnancies (at least one embryo in each uterine horn): 54.4% versus 45.6%. Although the multiple pregnancy rate was practically the same for the warm (26.5%) and cool period (26.3%), the difference between unilateral and bilateral multiple pregnancies varied from 17% during the warm period to 3% during the cool period (Figure 2). This suggests that ovulation of one follicle in cows with bilateral co-dominant follicles was reduced under heat stress conditions. Thus, the fate of bilateral multiple pregnancies was compromised during the warm period to the same extent as cooling of one follicle favored cooling of its neighbor co-dominant follicle in unilateral multiple pregnancies [53].

## 4. Conclusions

Multiple ovulations are the result of the simultaneous formation of two or more co-dominant follicles either from one or both ovaries [54,55]. Probably, the time span of the follicular stimulant hormone (FSH) threshold before follicular deviation is enough to allow multiple ovulations in many cases [56,57]. However, the final process leading to the events of ovulation can be blocked under certain circumstances such as the development of stress. If the above sequence is correct, cows under stress conditions with a single pre-ovulatory follicle at estrus may experience ovulation failure, whereas in cows with two or more co-dominant follicles, of which at least one ovulates, ovulation failure of the remaining follicles is often beyond the control of clinicians. We here propose heat stress as a model of stress in a time in which global warming is already a serious threat to reproductive function in animals and humans [45]. Possibly, follicular cooling before ovulation is not only susceptible to heat stress but also to any other type of stress. Thus, the physiological pattern or natural conditions of the ratio between unilateral and bilateral multiple pregnancies should be close to one, as was noted during the cool period in the study described above, i.e., 1.07 (346/323). In contrast, the ratio 1.4 (269/192) recorded during the warm period resulted in a higher incidence of bilateral ovulation failure rather than an increased rate of unilateral ovulations [53]. The fact that we observed a significant increase (*p* < 0.0001) in the conception rate (28–34 days post-insemination) from 31.1% during the warm period to 37.5% during the cool period over the four years of study supports this idea [53]. These results have clinical implications for reproductive control programs in dairy cattle. Hence, when assessing the reproductive performance of a herd at any given time, a ratio between unilateral and bilateral multiple pregnancies close to one could be a good indicator of cow wellbeing.

Quantifying the number of follicles likely to undergo ovulation at insemination has been the subject of only a limited number of studies. The incidence of cows developing two or more co-dominant follicles is currently close to 50%, particularly in older cows, and improvements in nutrition and management practices related to high milk production will promote further follicular co-dominance [58]. Double ovulation has been often related to a higher fertility [6,10,51,52]. Therefore, a balance between preserving fertility and preventing the risk of multiple pregnancies must be a focal point of clinical research. However, the most immediate question that needs addressing is how to reduce the risk of twin pregnancies and twinning. To this end, the transfer of a single embryo or drainage without suction of co-dominant follicles are recently proposed strategies [59,60], whereas once a cow becomes pregnant, management of twin pregnancies has also been discussed [61]. The follow-up of multiple ovulations following insemination is mandatory for optimizing reproductive management in dairy cattle.

## References

[1] Mee J.F. (1991). Factors affecting the spontaneous twinning rate and the effect of twinning on calving problems in nine Irish dairy herds. Irish Vet. J..

[2] Kinsel M.L., Marsh W.E., Ruegg P.L., Etherington W.G. (1998). Risk factors for twinning in dairy cows. J. Dairy Sci..

[3] Andreu-Vázquez C., Garcia-Ispierto I., Ganau S., Fricke P.M., López-Gatius F. (2012). Effects of twinning on the subsequent reproductive performance and productive lifespan of high-producing dairy cows. Theriogenology.

[4] López-Gatius F., Andreu-Vázquez C., Mur-Novales R., Cabrera V.E., Hunter R.H.F. (2017). The dilemma of twin pregnancies in dairy cattle. A review of practical prospects. Livest. Sci..

[5] Johanson J.M., Berger P.J., Kirkpatrick B.W., Dentines M.R. (2001). Twinning rates for North American Holstein sires. J. Dairy Sci..

[6] Fricke P.M., Wiltbank M.C. (1999). Effect of milk production on the incidence of double ovulation in dairy cows. Theriogenology.

[7] Lopez H., Caraviello D.Z., Satter L.D., Fricke P.M., Wiltbank M.C. (2005). Relationship between level of milk production and multiple ovulations in lactating dairy cows. J. Dairy Sci..

[8] López-Gatius F., López-Béjar M., Fenech M., Hunter R.H.F. (2005). Ovulation failure and double ovulation in dairy cattle: Risk factors and effects. Theriogenology.

[9] Garcia-Ispierto I., López-Gatius F. (2013). A three-day PGF_2α_ plus eCG-based fixed-time AI protocol improves fertility compared with spontaneous estrus in dairy cows with silent ovulation. J. Reprod. Dev..

[10] Garcia-Ispierto I., Roselló M.A., De Rensis F., López-Gatius F. (2013). A five-day progesterone plus eCG-based fixed-time AI protocol improves fertility over spontaneous estrus in high-producing dairy cows under heat stress. J. Reprod. Dev..

[11] Wiltbank M.C., Fricke P.M., Sangritasvong S., Sartori R., Ginther O.J. (2000). Mechanisms that prevent and produce double ovulations in dairy cattle. J. Dairy Sci..

[12] Andreu-Vázquez C., Garcia-Ispierto I., López-Gatius F. (2012). Photoperiod length and the estrus synchronization protocol used before AI affect the twin pregnancy rate in dairy cattle. Theriogenology.

[13] Labhsetwar A.P., Tyler W.J., Casida L.E. (1963). Analysis of variation in some factors affecting multiple ovulations in Holstein cattle. J. Dairy Sci..

[14] Macmillan K., Kastelic J.P., Colazo M.G. (2018). Update on multiple ovulations in dairy cattle. Animals.

[15] Hunter R.H.F., Einer-Jensen N., Greve T. (2006). Presence and significance of temperature gradients among different ovarian tissues. Microsc. Res. Tech..

[16] Hunter R.H.F. (2012). Temperature gradients in female reproductive tissues. Reprod. BioMed. Online.

[17] Hunter R.H.F., López-Gatius F., López-Albors O. (2017). Temperature gradients in vivo influence maturing male and female gametes in mammals: Evidence from the cow. Reprod. Fertil. Dev..

[18] Ng K.Y.B., Mingels R., Morgan H., Macklon N., Cheong Y. (2018). In vivo oxygen, temperature and pH dynamics in the female reproductive tract and their importance in human conception: A systematic review. Hum. Reprod. Update.

[19] Hunter R.H.F., López-Gatius F. (2020). Temperature gradients in the mammalian ovary and genital tract: A clinical perspective. Eur. J. Obstet. Gynecol. Reprod. Biol..

[20] Ali H.E., Kitahara G., Tamura Y., Kobayashi I., Hemmi K., Torisu S., Sameshima H., Horii Y., Zaabel S., Kamimura S. (2013). Presence of a temperature gradient among genital tract portions and the thermal changes within these portions over the estrous cycle in beef cows. J. Reprod. Dev..

[21] Hunter R.H.F., Nichol R., Crabtree S.M. (1980). Transport of spermatozoa in the ewe: Timing of the establishment of a functional population in the oviduct. Reprod. Nutr. Dev..

[22] Hunter R.H.F. (1981). Sperm transport and reservoirs in the pig oviduct in relation to the time of ovulation. J. Reprod. Fertil..

[23] Hunter R.H.F., Wilmut I. (1984). Sperm transport in the cow: Periovulatory redistribution of viable cells within the oviduct. Reprod. Nutr. Dev..

[24] Hunter R.H.F., Nichol R.A. (1986). A preovulatory temperature gradient between the isthmus and ampulla of pig oviducts during the phase of sperm storage. J. Reprod. Fertil..

[25] Hunter R.H.F., López-Gatius F. (2020). Evolutionary sequences in mammalian reproductive biology. J. Exp. Zool. A.

[26] Rutllant J., López-Béjar M., López-Gatius F. (2005). Ultrastructural and rheological properties of bovine vaginal fluid and its relation to sperm motility and fertilization: A review. Reprod. Domest. Anim..

[27] Hunter R.H.F., López-Gatius F. (2020). Intra-follicular temperature acts to regulate mammalian ovulation. Acta Obstet. Gynecol. Scand..

[28] Benoit H.J., Borth R., Ellicott A.R., Woolever C.A. (1976). Periovulatory changes in ovarian temperature in ewes. Am. J. Obstet. Gynecol..

[29] Grinsted J., Glendstrup K., Andreasen M.P., Byskov A.G. (1980). Temperature measurements of rabbit antral follicles. J. Reprod. Fertil..

[30] Grinsted J., Kjer J.J., Blendstrup K., Pedersen J.F. (1985). Is low temperature of the follicular fluid prior to ovulation necessary for normal oocyte development?. Fertil. Steril..

[31] Hunter R.H.F., Grondahl C., Greve T., Schmidt M. (1997). Graafian follicles are cooler than neighbouring ovarian tissues and deep rectal temperatures. Hum. Reprod..

[32] Hunter R.H.F., Bogh I.B., Einer-Jensen N., Müller S., Greve T. (2000). Pre-ovulatory Graafian follicles are cooler than neighbouring stroma in pig ovaries. Hum. Reprod..

[33] Greve T., Grøndahl C., Schmidt M., Hunter R.H.F., Avery B. (1996). Bovine preovulatory follicular temperature: Implications for in vitro production of embryos. Archiv. Tierzucht..

[34] López-Gatius F., Hunter R.H.F. (2017). Clinical relevance of pre-ovulatory follicular temperature in heat-stressed lactating dairy cows. Reprod. Domest. Anim..

[35] López-Gatius F., Hunter R.H.F. (2019). Pre-ovulatory follicular temperature in bi-ovular cows. J. Reprod. Dev..

[36] López-Gatius F., Hunter R.H.F. (2019). Pre-ovulatory follicular cooling correlates positively with the potential for pregnancy in dairy cows: Implications for human IVF. J. Gyn. Obstet. Hum. Reprod..

[37] Morita Y., Ozaki R., Mukaiyama A., Sasaki T., Tatebyashi R., Morishima A., Kitagawa Y., Suzumura R., Abe R., Tsukamura H. (2020). Establishment of long-term chronic recording technique of in vivo ovarian parenchymal temperature in Japanese Black cows. J. Reprod. Dev..

[38] Einer-Jensen N., Hunter R.H.F. (2000). Physiological and pharmacological aspects of local transfer of substances in the ovarian adnexa in women. Hum. Reprod. Update.

[39] Cicinelli E., Einer-Jensen N., Barba B., Luisi D., Alfonso R., Tartagni M. (2004). Blood to the corneal area of uterus is mainly supplied from the ovarian artery in the follicular phase and from the uterine artery in the luteal phase. Hum. Reprod..

[40] Einer-Jensen N., Hunter R.H.F. (2005). Counter-current transfer in reproductive biology. Reproduction.

[41] Roth Z. (2017). Effect of heat stress on reproduction in dairy cows: Insights into the cellular and molecular responses of the oocyte. Annu. Rev. Anim. Biosci..

[42] Hansen P.J. (2019). Reproductive physiology of the heat-stressed dairy cow: Implications for fertility and assisted reproduction. Anim. Reprod..

[43] Wolfenson D., Roth Z. (2019). Impact of heat stress on cow reproduction and fertility. Anim. Front..

[44] López-Gatius F., Hunter R.H.F. (2020). Local cooling of the ovary and its implications for heat stress effects on reproduction. Theriogenology.

[45] Boni R. (2019). Heat stress, a serious threat to reproductive function in animals and humans. Mol. Reprod. Dev..

[46] Garcia-Ispierto I., López-Gatius F. (2014). Effects of five-day progesterone-based fixed-time AI protocols on follicular/luteal dynamics and fertility in dairy cows. J. Reprod. Dev..

[47] Garcia-Ispierto I., De Rensis F., Pérez-Salas J.A., Nunes J.M., Pradés B., Serrano-Pérez B., López-Gatius F. (2019). The GnRH analogue dephereline given in a fixed-time AI protocol improves ovulation and embryo survival in dairy cows. Res. Vet. Sci..

[48] López-Gatius F., Garcia-Ispierto I., Serrano-Pérez B., Hunter R.H.F. (2018). The presence of two ovulatory follicles at timed artificial insemination influences the ovulatory response to GnRH in high-producing dairy cows. Theriogenology..

[49] Ginther O.J., Wiltbank M.C., Fricke P.M., Gibbons J.R., Kot K. (1996). Selection of the dominant follicle in cattle. Biol. Reprod..

[50] Garcia-Ispierto I., De Rensis F., Casas X., Caballero F., Mur-Novales R., López-Gatius F. (2018). Reproductive performance of lactating dairy cows after inducing ovulation using hCG in a five-day progesterone-based fixed-time AI protocol. Theriogenology.

[51] Bleach E.C., Glencross R.G., Knight P.G. (2004). Association between ovarian follicle development and pregnancy rates in dairy cows undergoing spontaneous oestrous cycles. Reproduction.

[52] Kusaka H., Miura H., Kikuchi M., Sakaguchi M. (2017). Incidence of double ovulation during the early postpartum period in lactating dairy cows. Theriogenology.

[53] López-Gatius F., Garcia-Ispierto I., Hunter R.H.F. (2020). Twin pregnancies in dairy cattle: Observations in a large herd of Holstein-Friesian dairy cows. Animals.

[54] Echternkamp S.E. (1992). Fetal development in cattle with multiple ovulations. J. Anim. Sci..

[55] Echternkamp S.E., Roberts A.J., Lunstra D.D., Wise T., Spicer L.J. (2004). Ovarian follicular development in cattle selected for twin ovulations and births. J. Anim. Sci..

[56] Acosta T.J., Beg M.A., Ginther O.J. (2005). Effects of modified FSH surges on follicle selection and codominance in heifers. Anim. Reprod..

[57] Scaramuzzi R.J., Baird D.T., Campbell B.K., Driancourt M.-A., Dupont J., Fortune J.E., Gilchrist R.B., Martin G.B., McNatty K.P., McNeilly A.S. (2011). Regulation of folliculogenesis and the determination of ovulation rate in ruminants. Reprod. Fertil. Dev..

[58] Vinet A., Drouilhet L. (2012). Genetic control of multiple births in low ovulating mammalian species. Mamm. Genome.

[59] López-Gatius F., Hunter R.H.F. (2019). Preventing twin pregnancies in dairy cattle, turning the odds into reality. Livest. Sci..

[60] López-Gatius F., Garcia-Ispierto I. (2020). Transfer of a single embryo versus drainage of subordinate follicles to prevent twin pregnancies in dairy cows. Why not both?. J. Reprod. Dev..

[61] López-Gatius F. (2020). Twins in dairy herds. Is it better to maintain or reduce a pregnancy?. Animals.

